# Small regulatory RNAs in *Streptococcus pneumoniae*: discovery and biological functions

**DOI:** 10.3389/fgene.2015.00126

**Published:** 2015-04-07

**Authors:** Joana Wilton, Paloma Acebo, Cristina Herranz, Alicia Gómez, Mónica Amblar

**Affiliations:** ^1^Unidad de Patología Molecular del Neumococo, Centro Nacional de Microbiología, Instituto de Salud Carlos IIIMadrid, Spain; ^2^CIBER Enfermedades RespiratoriasMadrid, Spain

**Keywords:** *Streptococcus pneumoniae*, virulence regulation, competence, small non-coding RNAs

## Abstract

*Streptococcus pneumoniae* is a prominent human pathogen responsible for many severe diseases and the leading cause of childhood mortality worldwide. The pneumococcus is remarkably adept at colonizing and infecting different niches in the human body, and its adaptation to dynamic host environment is a central aspect of its pathogenesis. In the last decade, increasing findings have evidenced small RNAs (sRNAs) as vital regulators in a number of important processes in bacteria. In *S. pneumoniae*, a small antisense RNA was first discovered in the pMV158 plasmid as a copy number regulator. More recently, genome-wide screens revealed that the pneumococcal genome also encodes multiple sRNAs, many of which have important roles in virulence while some are implicated in competence control. The knowledge of the sRNA-mediated regulation in pneumococcus remains very limited, and future research is needed for better understanding of functions and mechanisms. Here, we provide a comprehensive summary of the current knowledge on sRNAs from *S. pneumoniae,* focusing mainly on the *trans*-encoded sRNAs.

## Introduction

*Streptococcus pneumoniae*, the pneumococcus, is an opportunistic pathogen responsible for a wide spectrum of human diseases, ranging from mild otitis media to more severe infections such as meningitis, sepsis, or endocarditis. It is the main etiological agent of community-acquired pneumonia, causing more deaths in young children than any other infectious disease (O’Brien et al., 2009). Pneumococcal vaccines cover only a small number of the 93 different serotypes, and the treatment of pneumococcal diseases is hampered by the emergence and spread of drug-resistant strains^1^. *S. pneumoniae* is a normal component of the human commensal flora, asymptomatically colonizing the upper respiratory tracts of children and healthy adults. Human nasopharyngeal (NS) carriage is the source of transmission from person to person and serves as the first step in pathogenesis (Bogaert et al., 2004). Transition to opportunistic pathogen often occurs after a respiratory tract infection and is triggered by unknown host and bacterial factors. Disease progression exposes *S. pneumoniae* to numerous environmental changes and stress conditions, and rapid adaptation is a key factor for survival and replication.

In recent years, a plethora of RNAs with regulatory functions has been discovered in many pathogenic and non-pathogenic bacteria. These small RNAs (sRNAs) accomplish a large variety of regulatory functions, and are essential elements in bacterial pathogenicity (Toledo-Arana et al., 2007; Waters and Storz, 2009; Storz et al., 2011; Bobrovskyy and Vanderpool, 2013; Caldelari et al., 2013). Often non-coding, the sRNAs can act at the level of transcription, translation or RNA degradation. The majority of them regulate pathways that sense and transfer the external signals, and adapt the cell population in response to stress and environmental changes. Some regulate replication and maintenance of plasmids and phages (Brantl, 2002) and others, such as the CRISPR RNAs, protect the core genome from foreign nucleic acids (Fineran and Charpentier, 2012). They can act through three main mechanisms: (1) by base-pairing with nucleic acids, mostly mRNA targets, having either extensive or more limited complementarity; (2) modulating the activity of proteins by mimicking other nucleic acids, or (3) acting as riboswitches, sensing physical cues or metabolites and modulating expression of downstream genes (Winkler and Breaker, 2005; Zhang et al., 2010). RNA-interacting proteins play important roles in the expression and activity of sRNAs. Nucleases have critical roles in their production, quality control, and activation, and the RNA chaperone Hfq mediates the action of many sRNAs (Vogel and Luisi, 2011; Saramago et al., 2014).

Whereas a variety of sRNAs have been identified and studied in many Gram-positive and Gram-negative bacteria, little is known about these regulators in *S. pneumoniae*. Recent systematic approaches have uncovered multiple chromosomal-encoded sRNAs in the pneumococcus (Livny et al., 2006, 2008; Kumar et al., 2010; Tsui et al., 2010; Acebo et al., 2012; Mann et al., 2012) but only a few have been functionally studied (Halfmann et al., 2007; Schnorpfeil et al., 2013). In this review, we outline our current knowledge on the sRNA–mediated regulation in *S. pneumoniae*, compiling all information available and providing a comprehensive list of the sRNAs identified and their biological functions.

## RNAs Encoded in Extrachromosomal Elements

**FIGURE 1:**
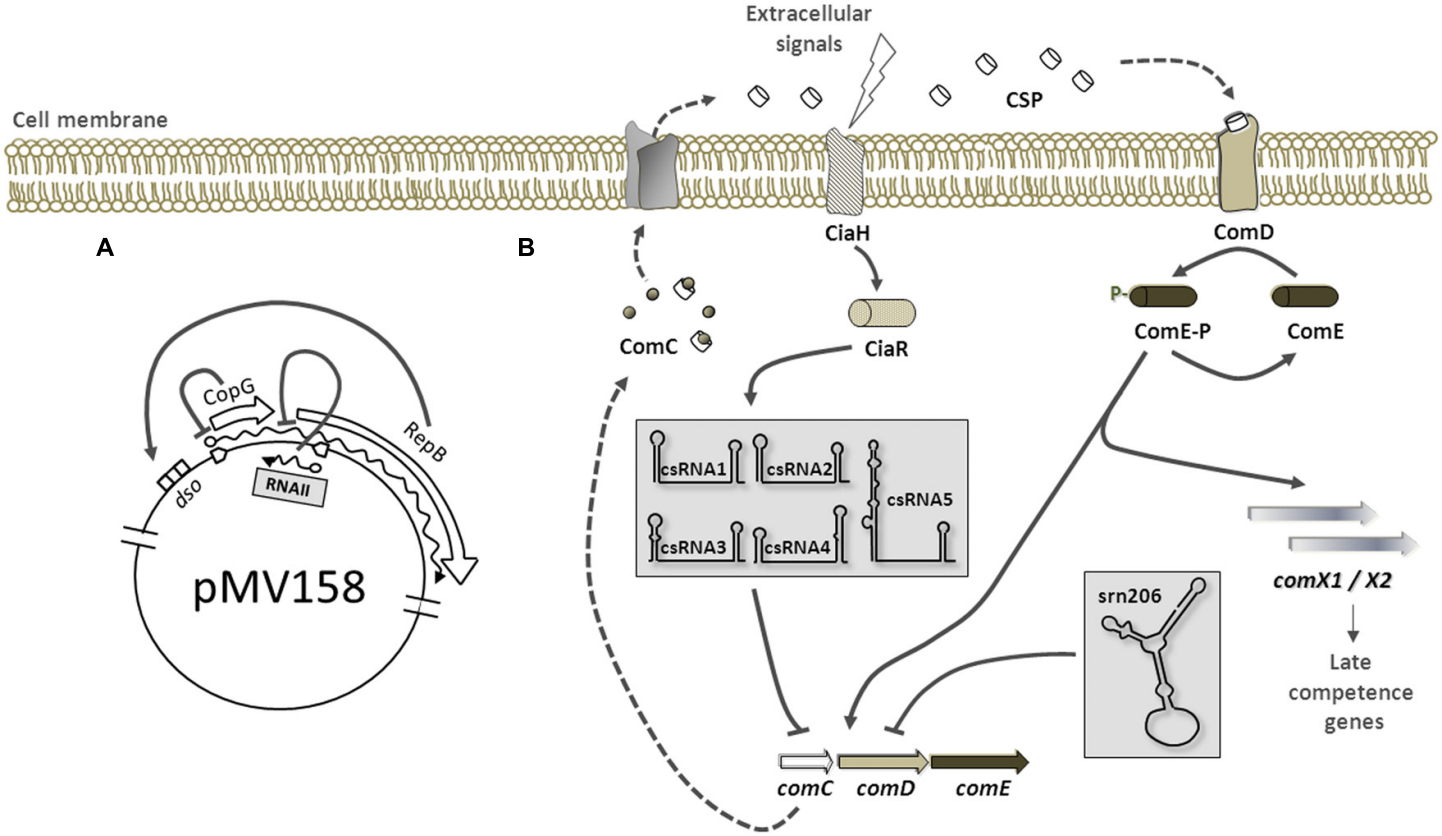
Regulation by small RNAs (sRNAs) in pneumococci. The cytosol and the extracellular environment (upper part of the figure) are separated by the cell membrane. **(A)** Replication of plasmid pMV158 is initiated by RepB protein upon binding to double stranded origin (dso). RepB transcription is inhibited by CopG. The antisense RNAII represses RepB translation by base-pairing with the region immediately upstream of RepB translational initiation signals in the *copG-repB* message. **(B)** Postulated mechanism of competence regulation by sRNAs. The extracellular concentration of CSP, an exported peptide pheromone derived from precursor protein ComC, is sensed by the membrane histidine kinase ComD. Binding of CSP to ComD results in phosphorylation of ComD, which then transfers the phosphate group to the cognate response regulator ComE, thus activating transcription of early competence genes (*comC, comD, comE, and comX1/X2*). *ComC*, *D* and *E* are cotranscribed in a long mRNA. Expression of the five csRNAs (within a gray box) is activated by the CiaRH two component system. The csRNAs then associate with the SD sequence and start codon of *comC* inhibiting its expression through an antisense mechanism. Similarly, the *srn206* (within a gray box) associates with *comD* message sequestering its translation initiation signals. The five csRNAs and the *srn206* act together to maintain the competence switched off. Predicted secondary structure of the csRNAs (by Mfold) and the *srn206* (by RNAfold) as previously published in (Halfmann et al., 2007) and (Acebo et al., 2012), respectively, is shown.

The first regulatory RNA discovered in pneumococcus was a plasmid-encoded *bona-fide* antisense RNA described by del Solar and Espinosa (1992), and its role in establishment, replication, and copy number regulation has been deeply investigated. The pMV158 is a promiscuous plasmid able to replicate in pneumococci, whose replication is initiated by the plasmid-encoded initiator protein RepB. Expression of RepB is subjected to a tight control exerted by two *trans*-acting plasmid elements, a transcriptional repressor protein (CopG) and an antisense RNA (RNAII; del Solar et al., 1995; **Figure 1A**). Both CopG and RepB are transcriptionally but not translationally coupled (López-Aguilar et al., 2013), and CopG is able to bind to their operator sequence and repress synthesis of the *copG-repB* mRNA (Hernández-Arriaga et al., 2009). Post-transcriptionally, the short 48-nt long antisense RNAII, whose synthesis is directed by the P_ctII_ promoter, inhibits translation of *repB* message by directly pairing to the region immediately upstream of its translational initiation signals (del Solar et al., 1997). Structural analyses by chemical and enzymatic probing, revealed that the RNAII consists of single stranded 5′ and 3′ tails and a hairpin, which together with the adjacent U-reach 3′ tail compose a very efficient intrinsic terminator (del Solar and Espinosa, 2001; López-Aguilar and del Solar, 2013). The most recent investigations (López-Aguilar, personal communication) demonstrated that the 5′-tail of RNAII play a critical role in the binding and translation inhibition of *repB* message, while the hairpin plays a secondary role. A singular binding mechanism is envisaged whereby initial pairing between complementary single stranded regions in the antisense and sense RNAs progresses upward into the corresponding hairpin to form the intermolecular duplex.

Both regulatory elements, CopG and RNAII, acts synergistically to ensure the plasmid copy number within a narrow range. The mechanism of repression by CopG has been extensively studied, as well as the RNAII mode of action, and constitutes the only regulatory RNA mechanistically studied in *S. pneumoniae*.

## Chromosomal-Encoded sRNAs

A combination of computational predictions, transcriptome analyses, and RNA sequencing approaches has been applied to discover chromosome-encoded riboregulators in *S. pneumoniae*. Although the available software for sRNA identification were mainly developed on the basis of sRNA features from Gram-negative genomes, adaptation of novel programs have also enabled the discovery of a large number of sRNAs in Gram-positive bacteria. As much as 128 sRNAs were bioinformatically predicted in intergenic regions (IGRs) of the pneumococcal chromosome through two different computational approaches, using the sRNAPredict2 software and the high throughput kingdom-wide prediction tool SIPHT (Livny et al., 2006, 2008). Additionally, searching for promoters containing a consensus CiaR (response regulator)-binding sequence allowed to identify the first class of five sRNAs located within IGRs of the genome (Halfmann et al., 2007). After that, three groups reported the use of high-throughput methodologies to experimentally discover sRNAs in pneumococci. First, Kumar and coworkers found 50 putative sRNAs in the clinical isolate TIGR4 genome by high-resolution tiling microarrays (Kumar et al., 2010). Further, Acebo and coworkers identified 88 potential sRNAs by deep sequencing, 68 of which were novel candidates (Acebo et al., 2012); and, finally, Mann et al. (2012) increased the total number to 178, of which 37 were identified by at least two different methods. Differences inherent to the technique used and/or different sequence coverage may explain the moderate overlapping observed between searches. Most of these sRNAs are conserved among pneumococcal species and some (71) are also present in other bacteria, mainly in fellow streptococci (59). Interestingly, about 40% (70) appear to be unique to this human pathogen.

Among the sRNAs identified, some belonged to previously known families of *Cis*-acting RNAs like riboswitches or leader regions (i.e., pyr, TPP riboswitch, T-box). Others belonged to the so-called functional sRNAs, such as RNase P, the 6S RNA, or tmRNA, whose predicted biological activities and mechanism of action are based on the knowledge of sRNA orthologs in other bacterial species. Some sRNAs have been identified as BOX elements, mobile sequences exclusively found in pneumococci and closely related species, which are numerous and randomly distributed in IGRs (Knutsen et al., 2006; Croucher et al., 2011). They have the potential to form stable stem-loop structures and may affect expression levels of neighbor genes either by stabilizing mRNAs or serving as DNA-binding sites for regulatory proteins. However, the majority of the sRNAs identified could not be assigned to a functional family. Remarkably, no *Cis*-antisense or CRISPR RNAs have been identified so far, in what appears to be a pneumococcal singularity.

## Biological Functions of Chromosomal-Encoded sRNAs

Expression of eighty out of the 178 putative sRNAs was tested by northern-blot or qRT-PCR and 70 of them were successfully validated (Halfmann et al., 2007; Kumar et al., 2010; Tsui et al., 2010; Acebo et al., 2012; Mann et al., 2012). However, knowledge about their biological function remains very limited and, with a few exceptions, no clearly defined targets or regulatory mechanisms were determined so far. Chromosome-encoded sRNAs have been mainly linked to fine-tuning metabolic processes or stress adaptation, and this often results in the lack of severe phenotypes upon deletion or overexpression. In fact, three sRNAs were deeply investigated in the D39 pneumococcal strain using deletion mutants and overexpressing strains, but no effects in common phenotypes or transcription patterns were conclusively found (Tsui et al., 2010). Nevertheless, several groups have recently succeeded in attributing diverse functions to pneumococcal sRNAs and some of them have been shown to control various aspects of virulence and participate in important regulatory networks such as competence or autolysis.

### sRNAs Control Virulence

The importance of sRNAs in virulence was overlooked until recently, when the application of targeted genetic knockouts and Tn-seq transposon screening mutagenesis demonstrated that a significant portion of the pneumococcal sRNAs have important global and niche-specific roles in virulence (Mann et al., 2012). Measurements of relative fitness and competitive index of sRNA pneumococcal mutants generated by random transposon insertion in the nasopharynx, lungs, and bloodstream, rendered a comprehensive list of sRNA mutants with altered fitness. The majority showed defects in nasopharynx colonization, lung infection, or replication in bloodstream (**Table 1**), but a small number of mutants actually resulted in a fitness benefit in certain host sites. These data indicate that sRNAs contribute to pneumococcal pathogenesis, both for systemic infections as well as for tissue-specific tropisms. Targeted knockout mutants validated these results and demonstrated that eight of them were also attenuated in establishing invasive disease upon intranasal (IN) challenge. These attenuated mutants were deeply studied, including analysis of their ability to adhere to and invade endothelial (ET) and NS cells, as well as transcriptomic and proteomic analyses to identify putative targets (for details see **Table 1**).

**Table 1:** Studied small RNAs (sRNAs) in pneumococci.

sRNA name	Pathogenesis profile^a^	Direct target and mechanism of action	Regulatory functions
RNAII^∗^		Translational repression of *repB* by base-pairing^b^	Control of plasmid replication^e^
F14	Nasopharynx		
F24	Nasopharynx		
F38; *srn254*; *spd-sr17*	Nasopharynx		
F51	Nasopharynx		
F52	Nasopharynx		
F63	Nasopharynx		
F64	Nasopharynx		
R16	Nasopharynx		
SN30	Nasopharynx		
SN39	Nasopharynx		
SN50	Nasopharynx		
*srn142*	Nasopharynx		FMN riboswitch
*trn0156*	Nasopharynx		
*trn0760*	Nasopharynx		
F66; *srn502;* SN27	Nasopharynx and blood		
R12; *trn0830*	Nasopharynx and blood		
R8	Nasopharynx and blood		
*trn1025*; SN46	Nasopharynx, blood, and lungs		
F41; *srn277*	Nasopharynx and blood; IN Challenge		
F20*; srn157*	Nasopharynx and lung; IN Challenge; reduced ET and NS adhesion and invasion		
F2	Blood		
F27; *trn0358*	Blood		TPP riboswitch
F45	Blood		
R4	Blood		
SN38	Blood		
*srn279*	Blood		
F5; *trn0052*	Blood and lungs		
*trn0012*; SN1; csRNA3	Blood and lungs	Translational repression of *comC*, *spr0081*, *spr0159*, *brnQ* and *spr1097*, by base-pairing^c^	Competence modulation and autolysis^f^
F25; *trn0332*; SN11	Blood; IN Challenge; reduced ET adhesion		
F26; SN12	Lung		Pyr regulator
F29	Lung		
F47; *srn368*; SN24	Lung		T-box
F59; *srn235*; SN20	Lung		
F60; *trn0485*	Lung		
F62	Lung		
R14	Lung		
R6; *srn400*	Lung		T-box
F8; SN5; csRNA1; *spd-sr56*	Lung	Translational repression of *comC*, *spr0081*, *spr0159*, *brnQ* and *spr1097*, by base-pairing^c^	Competence modulation and autolysis^f,g^
SN6; csRNA2	Lung	Translational repression of *comC*, *spr0081*, *spr0159*, *brnQ* and *spr1097*, by base-pairing^c^	Competence modulation and autolysis^f^
SN2	Lung		
SN22	Lung; reduced ET adhesion		
SN26	Lung		
SN31	Lung		
SN32	Lung		
*srn218*	Lung		
*trn0634*	Lung		
F32; *srn226*; SN16; tmRNA	Lung; IN Challenge; reduced ET and NS adhesion and invasion		*Trans*-translation^h^
F7; *srn061*; SN35; csRNA5	Lung; IN Challenge; reduced ET adhesion	Translational repression of *comC*, *spr0081*, *spr0159*, *brnQ* and *spr1097*, by base-pairing^c^	Competence modulation and autolysis^f^
F22	IN Challenge		
F44	IN Challenge; reduced ET adhesion		
F48	IN Challenge		
*srn395*; SN34; 6S			Control of gene expression in stationary phase^h^
*srn098*; SN8; RNaseP			tRNA maturation^h^
SN7; csRNA4		Translational repression of *comC*, *spr0081*, *spr0159*, *brnQ* and *spr1097*, by base-pairing^c^	Competence modulation and autolysis^f^
*srn206*		Translational repression of *comC*, *spr0081*, *spr0159*, *brnQ* and *spr1097*, by base-pairing^d^	Competence modulation^d^

*^∗^Plasmid encoded antisense RNA*.

*^a^Shows the contribution of sRNAs to pathogenesis on each host site as described in Mann et al. (2012). Deletion mutants with altered fitness in nasopharynx colonization, blood replication or lungs are indicated. Deletion mutants attenuated in invasive disease upon intranasal challenge (IN Challenge) or with reduced ability to adhere or to invade endothelial (ET) or nasopharyngeal (NS) cells are also indicated*.

*^b^del Solar et al. (1997); ^c^Schnorpfeil et al. (2013); ^d^Acebo et al. (2012); ^e^del Solar et al. (1995); ^f^Halfmann et al. (2007); ^g^Tsui et al. (2010); ^h^Biological activities predicted based on the knowledge in other bacteria*.

Two sRNAs of especial interest resulted from this extensive analysis: the F20, also named as *srn157*, and the F32, previously identified as the tmRNA (Kumar et al., 2010; Acebo et al., 2012). Both the *srn157* and tmRNA deletion mutants showed decreased adhesion/invasion of NS or ET cells, respectively, in concert with a lack of fitness and competitive index in the nasopharynx and lungs (**Table 1**). They also resulted in a dramatic alteration in abundance of several proteins (88 and 100, respectively), as well as in a substantial change in the gene expression profile. In case of *srn157* deletion mutant, proteomic analysis indicated that proteins responsible for purine metabolism were strongly downregulated, whereas DNA synthesis and repair pathways were greatly upregulated. Thus, deletion of this sRNA had pleiotropic effects that could explain its attenuation. In the tmRNA mutant, several metabolic networks encompassing the lactose transport system and multiple PTS systems were downregulated. The tmRNA has been associated with deficiencies in stress-response and pathogenicity in other bacteria (Withey and Friedman, 2003; Okan et al., 2006, 2010; Keiler, 2008) and has a central role in the *trans*-translation mechanism, a RNA and protein quality control system that resolves challenges associated with stalled ribosomes on non-stop mRNAs (Giudice et al., 2014; Shimizu, 2014). This role is consistent with the strong effect in pathogenesis observed in the tmRNA mutant.

All these data provide compelling evidence that sRNAs play important roles in virulence, that their effects can arise at several levels of control, and hence these roles can be restricted to specific host tissues. However, no direct regulatory link was established yet between sRNAs and putative targets.

### sRNAs Modulate Competence

Competence is a pivotal mechanism in *S. pneumoniae*, which regulate the expression of ∼200 genes and is involved in virulence and antibiotic resistance (Lau et al., 2001; Oggioni et al., 2004; Prudhomme et al., 2006; Kowalko and Sebert, 2008). But simultaneously, competence induction is highly stressful for the cell and needs to be tightly controlled. Thus, different layers of regulation in which proteins and regulatory RNAs act in concert to fine-tuning competence activation could be expected.

The first chromosomal-encoded sRNAs described in pneumococcus are part of the regulon of the two-component system CiaRH (Halfmann et al., 2007). These five csRNAs (cia-dependent small RNAs), designated from 1 to 5, are non-coding RNAs between 87 and 151 nt-long with a high degree of similarity to each other. Their predicted secondary structure consists of a stem-loop at the 5′-end and an unpaired region followed by a terminator stem-loop. Sequences complementary to the Shine-Dalgarno and the start codon AUG in the unpaired region suggested that csRNAs may control initiation of translation. CiaRH two-component system has been implicated in β-lactam susceptibility, autolysis, bacteriocin production, competence, and virulence, and some of these functions appear to be mediated by the csRNAs. For instance, stationary-phase autolysis was affected by csRNA4 and csRNA5 (Halfmann et al., 2007), and csRNA5 was defective in lung infectivity (Mann et al., 2012). But one of the most apparent phenotypes associated with CiaRH is blocking of spontaneous competence upon CiaRH activation (Guenzi et al., 1994; Müller et al., 2011). On this regard, csRNA1 was first shown to act negatively on competence development (Tsui et al., 2010), reversing the natural competence induction phenotype of the Δ*CiaRH* mutant, but no direct link between this csRNA and competence genes as targets could be established. Recently, csRNA target prediction analysis and evaluation by translational fusions identified six genes, which were all downregulated by the csRNAs acting additively (Schnorpfeil et al., 2013). One of these target genes was *comC*, encoding the precursor of the competence stimulating peptide CSP. Hyperactivation of CiaRH in the absence of csRNAs did not block competence development and partial disruption of *comC* complementarity to the csRNAs greatly diminished csRNA-mediated repression and relieved competence from CiaRH-dependent control. Therefore, CiaRH competence control is mediated by csRNAs, which block production of CSP precursor thereby inhibiting competence development (**Figure 1B**). Interestingly, CiaRH also controls production of the serine protease HtrA, which acts negatively on competence by degradation of CSP (Cassone et al., 2012). Which negative CiaRH-dependent control mechanism prevails, csRNA- or HtrA-mediated, depends strongly on growth conditions.

In addition to these five redundant csRNAs, another non-coding RNA, the *srn206*, has been suggested to participate in competence control. The *srn206* is a highly structured 120-nt long RNA that was predicted to associate to the translation initiation region of *comD* mRNA (Acebo et al., 2012). ComD is the histidine kinase of the ComDE two-component system that, upon induction by CSP, allows the entrance into a competent state of pneumococcal cells (Claverys et al., 2009; Johnston et al., 2014). Binding of CSP to ComD protein results in a phosphorylation cascade that finally activates the transcription of competence genes. Target prediction analysis suggested that *srn206* could regulate ComD levels by sequestering its start codon and ribosome-binding site, thereby preventing activation of competence (**Figure 1B**). In fact, overexpression of *srn206* significantly reduced the transformation efficiency of pneumococcal cells in response to exogenous CSP (Acebo et al., 2012). Nevertheless, no direct link between *srn206* and ComD translational repression could be established so far and more experiments are required to uncover the precise role of *srn206* in competence.

Therefore, although more investigation is required, current data suggests that different pneumococcal sRNAs may participate in competence modulation acting at distinct levels of the competence cascade, resembling the quorum sensing circuits described in other bacteria (Bejerano-Sagie and Xavier, 2007).

## Concluding Remarks and Perspectives

As shown above, numerous sRNAs have been identified in *S. pneumoniae*, but the understanding of sRNA-mediated regulation is largely insufficient and identification of targets and modes of action is still missing. Nevertheless, important aspects have been uncovered. For instance, the use of a multi-organ Tn-seq approach revealed that many sRNAs display global roles in discrete host tissues during disease, and provided a comprehensive list of sRNAs playing distinct roles in pathogenesis in the nasopharynx, the lung or the bloodstream. The analysis of sRNA contribution to pneumococcal pathogenesis in different host sites may provide a framework for future investigations to elucidate the precise function of these sRNAs. Moreover, a regulatory circuit including the concerted action of proteins and regulatory RNAs appears to control activation of competence in pneumococci. In this circuit, the five redundant csRNAs and the *srn206* act together, contributing to the maintenance of the competence on–off switch. Interestingly, all streptococcal genomes harbor from two to six csRNAs genes and their expression was validated for some of them (Marx et al., 2010). Therefore, the study of pneumococcal regulatory RNAs may uncover novel sRNA functions in other streptococcal species.

*Trans*-encoded antisense RNAs often require the action the RNA chaperone Hfq (Vogel and Luisi, 2011). This protein is present in 50% of all sequenced bacterial species, and a few species like *Bacillus anthracis* encode even two. In Gram-negative bacteria, Hfq is essential for activity and/or stability of most *trans*-encoded sRNAs. However, in Gram-positives its role is still controversial, and examples of Hfq-dependent antisense regulation have been reported only in *Listeria monocytogenes* (Nielsen et al., 2010) and *Clostridium difficile* (Boudry et al., 2014). As other streptococci, *S. pneumoniae* lacks an homolog of Hfq, and future research is needed to know whether pneumococcal *trans*-acting sRNAs require RNA chaperones to function or whether they have evolved fundamentally different mechanisms of action. Identification of a pneumococcal Hfq-like protein might be helpful to detect additional sRNAs and to identify targets.

The breadth of pneumococcal species and clinical isolates is an important issue for pneumococcal pathogenesis. They often differed in aspects such as invasiveness or antibiotic resistance, and comparison of their sRNA repertoire may help to elucidate their biological activity. Furthermore, the use of sRNAs as diagnostic tools and platforms for the development of antimicrobial therapies has long been suggested as an important outcome of sRNA studies. Clearly there are many exciting frontiers and unanswered questions in research on bacterial sRNAs and it is likely that important insights will come from breakthroughs in methodology. Understanding the ways that bacteria respond to and influence communities and how they survive such diverse environments will benefit from further studies of sRNAs.

## Footnotes

1 http://www.who.int/drugresistance/technicalguidance/en/resistantinfection.pdf
